# Effect of 1-year daily protein supplementation and physical exercise on muscle protein synthesis rate and muscle metabolome in healthy older Danes: a randomized controlled trial

**DOI:** 10.1007/s00394-023-03182-0

**Published:** 2023-06-02

**Authors:** Jacob Bülow, Bekzod Khakimov, Søren Reitelseder, Rasmus Bechshøft, Mikkel Jensen, Gerrit van Hall, Søren Balling Engelsen, Lars Holm

**Affiliations:** 1 Department of Orthopedic Surgery M, Institute of Sports Medicine Copenhagen M81, Bispebjerg Hospital, Building 8, Level 1, Nielsine Nielsens Vej 11, 2400 Copenhagen NV, Denmark; 2 Department of Food Sciences, University of Copenhagen, Copenhagen, Denmark; 3 Department of Biomedical Sciences, University of Copenhagen, Copenhagen, Denmark; 4 Clinical Metabolomics Core Facility, Department of Clinical Biochemistry, Rigshospitalet, Copenhagen, Denmark; 5 School of Sport, Exercise and Rehabilitation Sciences, University of Birmingham, Birmingham, UK

**Keywords:** Muscle metabolome, Protein synthesis, Aging, Protein supplementation, Training, Sarcopenia, Healthy older adults, Healthy aging

## Abstract

**Background:**

The skeletal muscle mass decreases with age and the responsiveness of aging muscles’ protein synthesis rate (MPS) to protein intake seems to deteriorate.

**Objective:**

This study investigated the impact of 12 months of protein supplementation with or without physical exercise training on the basal and postprandial MPS and the skeletal muscle metabolome of healthy older Danes (> 65 years, 29 females/37 males).

**Methods:**

Subjects were randomized to follow one of five intervention groups: (1) carbohydrate, (2) collagen protein, (3) whey protein, (4) home-based light resistance training with whey protein, and (5) center-based heavy-load resistance training with whey protein. Before and after the intervention, a tracer infusion trial was conducted to measure basal and postprandial MPS in response to intake of a cocktail consisting of 20 g whey hydrolysate + 10 g glucose. In addition, the skeletal muscle metabolome was measured using gas chromatography–mass spectrometry (GC–MS) at basal state and 4 h after the intake of the cocktail.

**Results:**

One year of daily protein or carbohydrate supplementation did not alter the basal and protein-stimulated postprandial muscle protein synthesis rate or the muscle metabolome of healthy older Danes. Basal MPS (%/h) at baseline for all subjects were 0.0034 ± 0,011 (mean ± SD). In contrast to previous studies, no difference was observed in basal MPS between males and females (*p* = 0.75). With the developed untargeted GC–MS methodology, it was possible to detect and tentatively annotate > 70 metabolites from the human skeletal muscle samples.

**Conclusion:**

One year of protein supplementation in comparison to an isocaloric-control supplement seems to affect neither the MPS at basal or postprandial state nor the skeletal muscle metabolome.

**Clinical trial registry:**

Number: NCT02115698, clinicaltrials.gov/ct2/show/NCT02115698.

**Supplementary Information:**

The online version contains supplementary material available at 10.1007/s00394-023-03182-0.

## Introduction

Preventing sarcopenia [1] by maintaining a well-functioning muscle mass is vital in the quest to remain independent, prevent falling [2] and to sustain quality of life at old age [3].

Both protein intake and physical activity are crucial (among other things) for maintaining and improving muscle mass and when inadequate detrimental muscle loss will follow [4, 5]. Both intake of proteins and physical activity stimulate an enhanced muscle protein synthesis (MPS) rate [6, 7], which are seen as the main determinants for fluctuations in the net protein balance.

In combination, resistance exercises also enhance/prolong the postprandial increments in MPS for up to 48 h [8]. The interplay in the skeletal muscle between being exercised and the responsiveness to hyperaminoacidemia could therefore support a recommendation for their combination. However, biological systems are known to adapt when exposed continuously or repeatedly. Most studies measuring the postprandial/postexercise muscle protein synthesis have been conducted in non-adapted subjects acutely exposed to the interventions. Therefore, the long-term adaptation in the responsiveness of the MPS rate is so far unknown. We recently showed in older males that the whole-body turnover response [9] was impaired and the muscle protein synthesis response [10] was unchanged in the acute hours to a standardized meal when habituated for 3 weeks to a diet high in protein compared to a diet with recommended protein intake. This could indicate that the interplay between exercise and protein intake on MPS is a temporary response in the non-adapted muscle, occurring only in the absence of habituation and, hence, not reflective of the state and responsiveness in the longer perspective.

Further, the protein quality (measured as the digestible indispensable amino acid score [11]) seems to influence the response to intake. Recently, Oikawa et al. found a greater increase, acute as well as 6-d integrated muscle protein synthesis, in response to the intake of a whey supplement (high-quality protein) compared to a collagen supplement (low-quality protein) [12]. However, the long-term effects of protein quality with respect to muscle preservation are so far unknown.

Both protein synthetic and muscular contractile processes are energy demanding and assumedly affect the skeletal muscle metabolome. Knowledge on alterations in the metabolome of the skeletal muscle in humans is scarce, due to analytical and technical challenges [13] as well as invasive sampling and in turn limited access to skeletal muscle tissue [14–18]. Insight into the intramuscular metabolome once habituated to higher protein intakes and resistance exercise training would reveal knowledge on the metabolic challenges the muscular environment handles and help understand the habituation process better. Any supportive knowledge on possible links between long-term exposures to elevated protein intake alone and in combination with exercise training will get us closer to understanding the mechanisms and form the basis for formulating recommendations for older adults.

This study therefore aims to elucidate whether adaptation to long-term exposure to exercise training and/or enhanced protein intake affects the basal MPS as well as the responsiveness to a single serving of protein in healthy older individuals above 65 years.

The setting of the present study was a 12-month intervention with daily supplementation of high- or low-quality protein and carbohydrate as non-protein control, and two groups on the high-quality protein diet conducting either heavy or light load resistance training. We hypothesized that the basal and protein-stimulated postprandial MPS rates are elevated in the exercise training groups after 12 months of intervention and that the intake of protein of different qualities will not affect the acute muscle protein synthetic response to protein intake. The impact of the 12-month interventions and acute postprandial state on the skeletal muscle metabolome was explored with the purpose of generating hypotheses.

## Methods

The CALM trial (Counteracting Age-Related Loss of Muscle Mass) was conducted at Bispebjerg Hospital between 2014 and 2018. It was designed as an intention-to-treat randomized controlled study. The study reported in this paper was conducted on a subgroup of subjects, who in addition to the general measurements performed in the CALM trial also participated in an acute 1-day stable isotope tracer infusion trial before and after the intervention. The primary outcome of the study was the comparison of basal overnight fasted muscle protein synthesis rate as well as muscle protein synthesis rate response to protein intake from basal overnight fasted state between baseline and after 12 months. We further explored any sex-specific changes regarding the muscle protein synthesis rates as well as changes in the skeletal muscle metabolome. The exploratory analysis was not pre-specified.

Further information and detailed description of the purpose, methods and exclusion criteria in the CALM trial has been published previously [19]. The trial protocol (H-4-2013-070 and H-4-2013-070.3) was approved by the regional ethics committee and all participants provided written informed consent. The trial protocol for this study was registered at clinicaltrials.gov journal number: NCT02115698.

### Participants

This study includes 66 healthy older adults above 65 years of age. All participants were screened by a physician prior to enrollment. Participants were excluded if they performed > 1 h of heavy resistance training per week and if they had any medical condition potentially preventing them from completing the 1-year intervention. Participants were allowed to be medicated against hypertension, hypercholesterolemia and thyroid dysfunction. For an exact list of accepted medications see the trial protocol [19].

### Study design

After enrollment, participants were randomized using minimization (software MinimPy 0.3; http://minimpy.sourceforge.net/) and stratified by sex and number of completed repetitions on the 30-s chair stand test (< 16 or ≥ 16) into one of five intervention groups: (1) carbohydrate supplementation (CARB; 20 g maltodextrin + 10 g of sucrose), (2) collagen protein supplementation (COLL; 20 g bovine collagen protein hydrolysate (ATpro 200) + 10 g sucrose), (3) whey protein supplementation (WHEY; 20 g whey protein isolate (LACPRODAN, Arla Foods Ingredients P/S, Viby J, Denmark) + 10 g of sucrose), (4) heavy resistance training with whey protein supplementation (HRTW), (5) light-intensity training with whey protein supplementation (LITW). All groups were instructed to dissolve their respective powder-based supplement in water, juice or milk two times daily at breakfast and lunch. All supplements were developed and packaged by Arla Foods Ingredients Group P/S, Viby J, Denmark (for further information, see Bechshøft et al. [19]). The HRTW group followed a supervised center-based progressive heavy resistance exercise program three times weekly and the LITW group was instructed to do a home-based non-supervised progressive light-load resistance training program three to five times weekly using TheraBand^®^ rubber bands (Hygenic Corp., Akron, OH, USA) and bodyweight. For further details, see prior publication [20]. Before and after the 1-year intervention, all participants went through a thorough battery of tests (see previous publication [19]) and an acute stable isotope tracer infusion trial. The present paper primarily reports results from these acute trials (see below).

### Acute trial

**Fig. 1:**
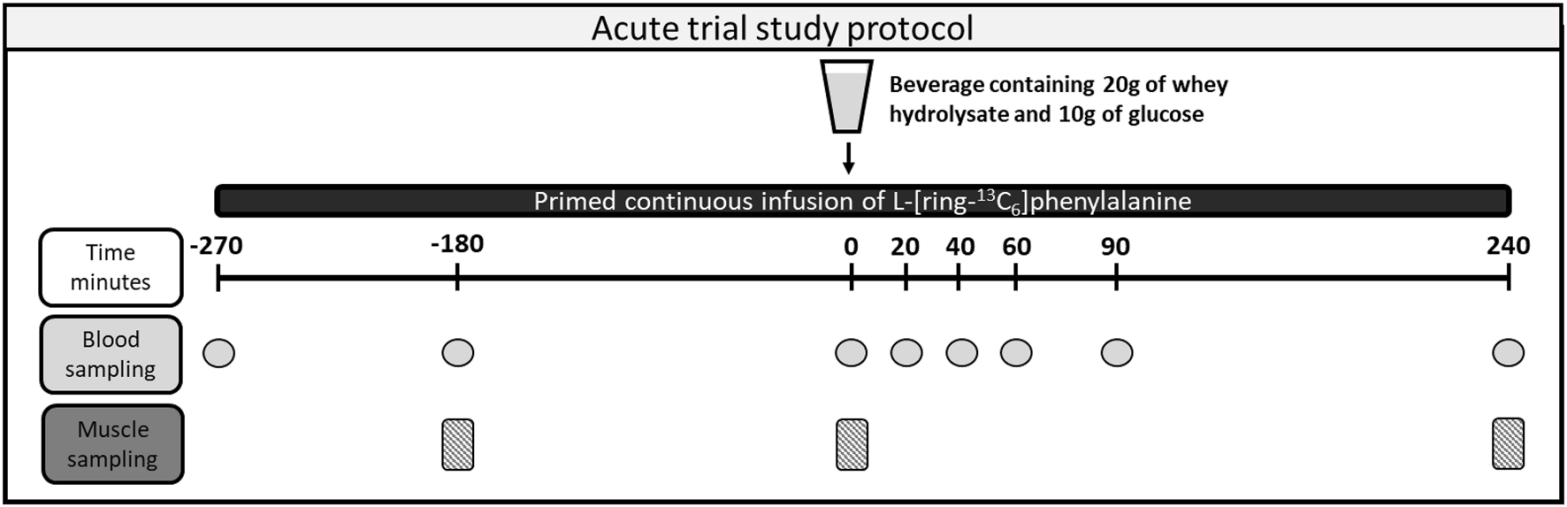
Acute trial study protocol conducted at before and after the 12-month of intervention period

Participants arrived at the facility 8 a.m. in the morning by car or public transportation to avoid physical activity in an overnight fasted state from 9 p.m. the day before. They were instructed to abstain from strenuous physical activity 3 days prior to the trial. The participants were placed in a bed in a supine position and two venous catheters were inserted in an antecubital vein in each arm and a background blood sample was taken. Hereafter, at − 270 min (see Fig. 1), a continuous infusion with L-[^13^C_6_] phenylalanine tracer (Cambridge Isotope Laboratories, Tewksbury, MA, USA) at an infusion rate of 6.0 µmol kg FFM^−1^ h^−1^ was started after injection of a priming dose 6.0 µmol kg FFM^−1^ over 2 min. The tracers were dissolved in sterile saline water and filtered through 0.20-µm-pore disposal filters (Minisart, Sartorius Stedium Biotech, Gottingen, Germany). The tracer infusion rate was set to obtain a venous tracer-to-tracee ratio (TTR) of ~ 10%. After reaching steady state at − 180 min, another blood sample and the first muscle biopsy were taken. The participants continued to rest in the supine position until another blood sample and biopsy were taken at 0 min. Immediately after, a drink containing 20 g of whey hydrolysate and 10 g of glucose was provided and finished immediately. Then blood samples were taken at 20 min, 40 min, 60 min, 90 min, 120 min and 240 min after the biopsy. At 240 min the last biopsy was taken, and the infusion stopped.

### Blood samples

All blood samples were collected in 9 mL plasma Vacutainers containing EDTA, placed on ice for ≥ 10 min, and spun down at 3200 g for 10 min at 4 °C. Plasma was then transferred to Eppendorf tubes and stored at − 80 °C until further analysis.

### Muscle biopsies

All three biopsies were obtained from the vastus lateralis with individual incisions with ~ 3 cm in between with a 4-mm biopsy needle (Bergström, Stockholm, Sweden) using manual suction. At the beginning of the trial, the skin was shaved, the thigh muscle was inspected and the incision sites for the three biopsies were marked. Before obtaining each biopsy, the area was disinfected and local anesthetic (1% lidocaine) was administered. A ~ 1 cm incision was made before inserting the needle and obtaining the biopsy. An elastic band with a compression pad was used to compress the incision site for 30 min to avoid intramuscular hematoma. Before compression, the incisions were strapped with SteaStrips and covered with waterproof plaster. The muscle specimens were quickly cleansed from any visible blood, fat and connective tissue under a microscope, and then frozen in liquid N_2_ and stored at − 80 °C until further analysis.

### FSR

The myofibrillar protein fractional synthesis rate (FSR) was calculated for two periods (see Fig. 1) using the precursor–product model as illustrated:

$$\mathrm{FSR}=\frac{{\Delta \mathrm{E}}_{\mathrm{Myofibrilar protein},\mathrm{ Phe}}}{({\mathrm{E}}_{\mathrm{Plasma mean},\mathrm{ Phe}} \times t)} \times 100,$$

where ${\Delta \mathrm{E}}_{\mathrm{Myofibrilar protein},\mathrm{ Phe}}$ is the change in myofibrillar protein-bound phenylalanine enrichment between two consecutive biopsies with *t* hours in between; ${\mathrm{E}}_{\mathrm{Plasma mean},\mathrm{ Phe}}$ is the plasma weighted mean phenylalanine enrichment between the two biopsies. The 3-h basal synthesis rate was calculated using the biopsies and blood samples at − 180 min and 0 min (abbreviated FSR_basal_), and the 4-h synthesis rate in response to protein intake using the biopsies at 0 min and 240 min and a weighed mean of the plasma enrichment levels measured in the blood samples from 0, 20, 60, 90 and 240 min (abbreviated FSR_response_). A factor of 100 was used to express FSR in percent per hour (% h^−1^) [21]. The muscle specimens were prepared as follows: ~ 20 mg of the muscle sample was transferred to a 2-mL lysing tube containing 10 lysing beads and two silicon carbide crystals. One mL of 4 °C homogenizing buffer (Tris 0.02 M [pH 7.4], NaCl 0.15 M, ED(G)TA 2 mM, Triton X-100 0.5%, sucrose 0.25 M) was added and the sample was homogenized 4 ∙ 45 s at a speed of 5.5 m s^−1^ with a 2-min pause in between (FastPrep 120A-230; Thermo Savant, Holbrook, NY, USA). The samples were then rested for 3 h at 5 °C. They were then spun at 800 g for 20 min at 5 °C and the supernatant discarded. One mL of 4 °C homogenizing buffer were added to the pellet and the sample was once again homogenized for 1 ∙ 45 s at a speed of 5.5 m s^−1^, left for 30 min at 5 °C and then spun 800 g for 20 min at 5 °C. The supernatant was again discarded and 1.5 mL KCl buffer (KCl 0.7 M, pyrophosphate (Na_4_P_2_O_7_) 0.1 M) added and the samples were vortexed and left overnight at 5 °C. The sample was then vortexed and spun at 1600 g for 20 min at 5 °C and the supernatant (the myofibrillar protein fraction) was then transferred to a Scot glass and 2.3 mL ethanol 99% was added. The samples were then vortexed and left for 2 h at 5 °C. After a spin at 1600 g for 20 min at 5 °C, the supernatant was discarded and 1 mL 70% ethanol was added to the pellet containing the myofibrillar protein fraction. The samples were vortexed and then spun at 1600 g for 20 min at 5 °C and the supernatants were once again discarded. To hydrolyze the myofibrillar proteins, 1 mL of 6 M HCl was added and the sample left overnight at 110 °C. The constituent amino acids were then purified over Dowex resin (AG 50W-X8 resin; Bio-Rad Laboratories, Hercules, CA) columns using 2 M NH_4_OH for elution and put under N_2_ flow at 70 °C until dried. Hereafter, the amino acids were derivatized as the N-acetyl-propyl (NAP) derivative as described in detail previously [22]. After derivatization, the samples were analyzed using a gas chromatography combustion isotope ratio mass spectrometry (GC–C–IRMS) system (Hewlett Packard 5890-Finnigan GC combustion III-Finnigan Deltaplus; Finnigan MAT; Bremen; Germany). Briefly, 1 µL of sample was injected using a solvent split mode programmed-temperature vaporization (PVT) inlet. A detailed description of settings has been published previously [21]. The tracer enrichments in plasma were analyzed using liquid chromatography–tandem mass spectrometry (LC–MS/MS). Plasma samples were prepared and analyzed as described by Bornø et al. 2014 [23].

### Muscle metabolome

The muscle metabolome was measured using biopsies at time point 0 min and 240 min after the intake of cocktail, both at 0 month and after 12 months of intervention. Muscle samples were extracted using a similar method as described by Alves et al. 2015 [16], which is based on methanol/chloroform/water at Vol:Vol:Vol ratio of 1:1.2:1, respectively. The muscle specimens were prepared and analyzed as follows. ~ 25 mg of frozen muscle tissue was put into 2 mL lysing tubes containing 10 lysing beads (MP Blomedicals, Ohio, USA) and two silicon carbide crystals (Biospech products inc., Bartlesville, USA). Then 0.5 mL of ice-cooled 50% methanol, containing 20 ppm ribitol, was added. The biopsies were homogenized by stirring four times with an interval of 1 min at a speed of 5.5 m s^−1^ at 5 °C (FastPrep 120A-230, Thermo Savant, Holbrook, NY, USA) with a 2 min pause in between to avoid heating. Then, 300 µL of chloroform was added and the homogenized samples were vigorously vortexed for 10 min at room temperature. The samples were rested on ice for 20 min and centrifuged for 15 min at 5 °C at 16,000 g. Sixty µL of the upper part of the aliquot (methanol part) and 40 µL of the lower part of the aliquot (chloroform part) were put into 200 µL glass inserts. The glass inserts were then dried under reduced vacuum using a SpeedVac (Labogene, Lynge, Denmark) at 40 °C for 3 h. Samples were then derivatized in two steps, first by addition of 10 µL 20 mg mL^−1^ methoxamine hydrochloride in dry pyridine (90 min at 45 °C by agitating at 750 rpm) followed by trimethylsilylation (TMS) using trimethylsilyl cyanide (TMSCN), as described previously [24]. TMS derivatization was performed by addition 40 µL TMSCN and by agitating at 750 rpm for 40 min at 45 °C. A total of 226 samples were analyzed by GC–MS in a randomized order; 206 samples originate from this study design and 20 samples were controls consisting of pooled muscle samples run every 10th sample in the sequence.

Sample derivatization and injection of 1 µL derivatized aliquot were automated using a Dual-Rail MultiPurpose Sampler (MPS) (Gerstel, Mülheim an der Ruhr, Germany) as previously described [25]. The GC–MS consisted of an Agilent 7890B gas chromatograph (GC) (Agilent Technologies, California, USA) coupled with a HT Pegasus time-of-flight mass spectrometer (LECO Corporation, Saint Joseph, USA). A GC column used was Restek ZB 5% Phenyl 95% Dimethylpolysiloxane column (30 m length, 25 µm diameter and 0.25 µm of film thickness) with a 5 m inactive guard column (Phenomenex, Torrance, USA). A hydrogen generator, Precision Hydrogen Trace 500 (Peak Scientific Instruments Ltd, Inchinnan, UK) was used to supply a carrier gas at the constant column flow rate of 1.0 mL min^−1^. The initial temperature of the GC oven was set to 40 °C and held for 2 min, followed by heating at 12 °C min^−1^ to 320 °C and kept for an additional 8 min, making the total run time 33.3 min. A post-run time at 40 °C was set to 5 min. Mass spectra were recorded in the range of 45–600 *m*/*z* with a scanning frequency of 10 Hz, and the MS detector and ion source were switched off during the first 6.4 min of solvent delay time. The transfer line and ion source temperature were set to 280 °C and 250 °C, respectively. The mass spectrometer was tuned according to manufacturer’s recommendation using perfluorotributylamine (PFTBA). The MPS and GC–MS were controlled using vendor software Maestro (Gerstel, Mülheim an der Ruhr, Germany) and ChromaTOF (LECO Corporation, Saint Joseph, USA). The raw GC–TOF–MS data were processed using Statistical Compare toolbox of the ChromaTOF software (Version 4.50.8.0) with the following settings: the raw data were used without smoothing prior to peak deconvolution; the baseline offset was set to 0.8; the expected average peak width was set to 1.2 s; the signal-to-noise was set to ≥ 5; the peak areas were calculated using deconvoluted mass spectra; the common m/z ions of derivatization products were determined as 73, 75, and 147. Deconvoluted mass spectra were also used for peak identification using LECO-Fiehn and NIST11 libraries. The library search was set to return top 10 hits with EI-MS match of > 75% using normal-forward search and with a mass threshold of 20. Deconvoluted peaks were aligned across all samples using the following settings: retention time shift allowance of < 3 s, EI-MS match > 90%, mass threshold > 25 and present in > 90% of all pooled samples.

### Statistical analysis

FSR were analyzed according to the published study protocol [19] with a one-way ANOVA on each intervention arm separately comparing the difference between delta fractional synthesis rates (the FSR in the resting 4-h postprandial period after ingestion of 20 g whey protein ‘response’ subtracted the resting overnight fasted FSR ‘basal’) at 0 month and 12 months (ΔΔ FSR = (^12month^FSR_response_ − ^12month^FSR_basal_) − (^0month^FSR_response_—^0month^FSR_basal_)). Further, two-way mixed ANOVA test was performed on the 0-month data set on basal vs. response with sex as the between-group factor. All FSR statistical analyses were performed using GraphPad Prism version 8.0.0 for Windows (GraphPad Software, San Diego, California, USA). FSR were analyzed as modified intention to treat (mITT) that completed the two trials irrespective of adherence and as per protocol (PP). To be included in the PP analysis, participants needed a supplement adherence > 75%, as well as > 65% and > 75% adherence to training for those assigned to HRTW and LITW, respectively. Further, participants who conducted the 12-month acute trial more than 14 days after their last training session were also excluded from the PP analysis. mITT analyses were conducted on participants who completed the study irrespective of adherence to the intervention. The muscle metabolome data were subjected to univariate and multivariate statistical analysis prior to investigating the possible effects according to the study design factors, including visit (0 m and 12 m), treatment (basal and response) and the intervention (CARB, COLL, WHEY, LITW and HRTW). Principal component analysis (PCA) [26] was performed prior to explore the muscle metabolome data and evaluate the overall systematic variation present in the data. ANOVA-simultaneous component analysis (ASCA) [27] with permutation test, as described previously [28], was used to study the significance of the study design factors and their explained variations. Single metabolite differences related to the design factors were analyzed using an ANOVA adjusted for multiple testing using false discovery rate (FDR) of 10%. Prior to PCA, ASCA and ANOVA, the muscle metabolome data were normalized to the internal standard (ribitol) peak area. The muscle metabolome data were mean centered (the mean of each peak was subtracted from the corresponding variable) and divided by its standard deviation, also called “auto scaling” prior to PCA and ASCA analysis. All data analyses were conducted using MATLAB ver. 2016b (The Mathworks, Inc. USA) and custom MATLAB scripts written by the authors.

### Blinding

Randomization was done by an investigator not involved in interventions or not sensitive to blinding. Participants in the WHEY, COLL and CARB group were blinded to which supplement they received. Training was not blinded to the participants. Blinded researchers performed and analyzed the outcome measures.

## Results

### Participants and compliance

Out of the 66 participants included, 29 were female and 37 were male with a mean age of 70 years [median: 69 years; range: 65–80 years]. Baseline subject characteristics for the five different intervention groups are presented in Table 1. Out of the 66 subjects, who participated in the acute trial at 0 month, 64 completed it. Two participants (1 COLL, 1 LITW) missed the 240-min biopsy due to complications during the trial at 0 month and they did not participate in the acute trial at 12 months. Further, nine subjects did not participate at 12 months (1 CARB, 3 COLL, 2 WHEY, 2 LITW, 1 HRTW) due to complications, discomfort after the acute trial at 0 month or general dropouts, resulting in 55 subjects with complete sample sets. In the HRTW group, six subjects had an adherence below 65%, two subjects had a supplementary adherence below 75%, and two subjects conducted their acute trial more than 14 days after the last training session, which was the time span set a priori. Thus, in the HRTW group, FSR and muscle metabolome data were available for only 3 out of the 11 subjects who completed both 0- and 12-month acute trial with sufficient compliance and time between the intervention stop and the acute trial to be included in a per-protocol (PP) analysis. The training and supplementary mean adherence for these three subjects were 80% ± 11% (mean ± SD) and 86% ± 10%, respectively. In the LITW group, four subjects had an adherence below 75%, two subjects had no training or supplementary registrations, and two subjects conducted their acute trial more than 14 days after the last training session. Thus, in the LITW group, FSR data were available on four subjects and the muscle metabolome data were available on three out of the nine subjects that had completed 0- and 12-month acute trial with sufficient compliance. The training and supplementary mean adherence for these four subjects were 86% ± 7% and 86% ± 5%, respectively. In the WHEY group, eight subjects completed the 0- m and 12-m acute trial with sufficient compliance to be included in a PP analysis for the FSR measurements and six for the muscle metabolome measurements. The supplementary mean adherence for these eight subjects was 94% ± 5%. In the COLL group, nine subjects completed the 0- and 12-month acute trial with sufficient adherence and were included in a PP analysis for the FSR measurements, and due to the amount of tissue sample the muscle metabolome was measured for six subjects. The supplementary mean adherence for these nine subjects was 91% ± 7%. In the CARB group, eight subjects completed the 0- and 12-month acute trial with sufficient adherence to be included in a PP analysis for the FSR measurements and six for the muscle metabolome measurements. The supplementary mean adherence for these eight subjects was 87% ± 9%.

**Table 1:** Subject characteristics baseline

Subject characteristics at baseline (0 m)	CARB	COLL	WHEY	LITW	HRTW	One-way ANOVA
*N* (males|females)	12 (6|6)	15 (8|7)	15 (9|6)	12 (6|6)	12 (8|4)	
Age (y)	69 ± 4	70 ± 4	71 ± 5	69 ± 3	69 ± 3	*P* = 0.71
Height (m)	1.70 ± 0.06	1.72 ± 0.07	1.74 ± 0.07	1.72 ± 0.09	1.74 ± 0.09	*P* = 0.66
Weight (kg)	74.3 ± 11.3	77.0 ± 11.4	70.7 ± 8.5	73.2 ± 9.8	78.1 ± 14.7	*P* = 0.43
BMI (kg m^2^)	25.6 ± 3.6	26.2 ± 3.5	23.3 ± 1.9	24.8 ± 2.4	25.5 ± 3.3	*P* = 0.12
Fat free mass (kg)	48.3 ± 7.2	49.6 ± 9.3	48.2 ± 8.0	47.7 ± 10.4	52.8 ± 10.4	*P* = 0.64
30 s chair rise test (repetitions)	21 ± 7	19 ± 4	19 ± 4	20 ± 4	21 ± 3	*P* = 0.74
Systolic blood pressure (mmHg)	154 ± 15	146 ± 24	145 ± 17	144 ± 19	149 ± 24	*P* = 0.74
Diastolic blood pressure (mmHg)	91 ± 10	82 ± 9	80 ± 9	85 ± 11	84 ± 9	*P* = 0.06
Glucose fasted (mmol L^−1^)	5.5 ± 0.5	5.6 ± 0.4	5.5 ± 0.5	5.4 ± 0.3	5.6 ± 0.5	*P* = 0.78
Glucose 2 h OGTT (mmol L^−1^)	6.8 ± 1.3	6.6 ± 1.5	6.1 ± 1.0	6.6 ± 1.1	6.0 ± 1.2	*P* = 0.40
Haemoglobin A1c (mmol mol^−1^)	36 ± 2	36 ± 4	35 ± 3	34 ± 3	36 ± 2	*P* = 0.35
Total cholesterol (mmol L^−1^)	5.9 ± 1.1	5.8 ± 1.3	5.7 ± 0.8	5.7 ± 0.9	5.9 ± 0.6	*P* = 0.97
HDL cholesterol (mmol L^−1^)	1.9 ± 0.5	1.9 ± 0.5	2.0 ± 0.6	1.8 ± 0.4	1.9 ± 0.5	*P* = 0.90
LDL cholesterol (mmol L^−1^)	3.4 ± 1.0	3.2 ± 1.2	3.1 ± 0.6	3.4 ± 1.0	3.5 ± 0.6	*P* = 0.78

Means ± SD

The CARB group protein intake did not change (− 4.9 g, 95% CI [− 15.8;6.1]). COLL,WHEY, LITW and HRTW groups significantly increased their protein intake by 29.0 g (95% CI [21.1;36.8]), 25.7 g (95% CI [15.6;35.8]), 23.9 g (95% CI [15.2;32.5]) and 26.7 g (95% CI [18.9;34.5]) corresponding to an approximal increase in total protein intake from ~ 1.1–1.5 g kg^−1^ pr. day[20].

For an overview, see CONSORT diagram in supplemental 5.

### FSR

The FSR_basal_ and FSR_response_ for the respective groups for all subjects completing the acute trial at 0 and 12 month were as follows (*mean* ± *SD; [%∙h*^*−1*^*])*:

CARB: ^0month^FSR_basal_ = 0.034 ± 0.009, ^12month^FSR_basal_ = 0.034 ± 0.009, ^0month^FSR_response_ = 0.039 ± 0.008, ^12month^FSR_response_ = 0.043 ± 0.013.

COLL: ^0month^FSR_basal_ = 0.034 ± 0.011, ^12month^FSR_basal_ = 0.029 ± 0.013, ^0month^FSR_response_ = 0.039 ± 0.012, ^12month^FSR_response_ = 0.037 ± 0.012.

WHEY: ^0month^FSR_basal_ = 0.033 ± 0.012, ^12month^FSR_basal_ = 0.031 ± 0.017, ^0month^FSR_response_ = 0.039 ± 0.012, ^12month^FSR_response_ = 0.033 ± 0.011.

LITW: ^0month^FSR_basal_ = 0.038 ± 0.012, ^12month^FSR_basal_ = 0.031 ± 0.012, ^0month^FSR_response_ = 0.042 ± 0.013, ^12month^FSR_response_ = 0.034 ± 0.011.

HRTW: ^0month^FSR_basal_ = 0.032 ± 0.012, ^12month^FSR_basal_ = 0.031 ± 0.021, ^0month^FSR_response_ = 0.036 ± 0.014, ^12month^FSR_response_ = 0.031 ± 0.012.

**Fig. 2:**
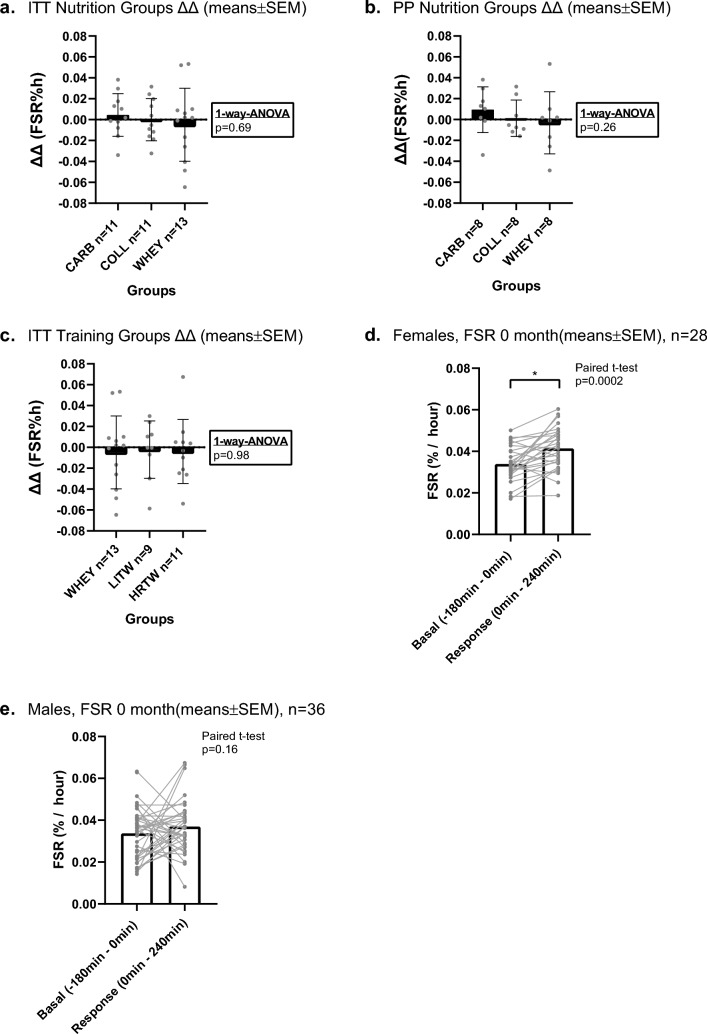
**a** mITT analysis of ΔΔ FSR in the nutritional arm, **b** PP analysis of ΔΔ FSR in the nutritional arm, **c** mITT analysis of ΔΔ FSR in the training arm, **d** Females basal vs. response FSR at 0 month, **e** Males basal vs. response FSR at 0 month. * denotes significant difference (*p* < 0.05). Boxes are means ± SEM for all plots

Comparing the ΔΔ (ΔΔ = (^12month^FSR_response_—^12month^FSR_basal_) − (^0month^FSR_response_—^0month^FSR_basal_) FSR between groups in the nutrition supplementation arm, no difference was observed irrespective of adherence to the intervention (mITT: *p* = 0.69; PP: *p* = 0.26) (m*ITT, mean[%∙h*^*−1*^*]* ± *SEM:* CARB 0.0045 ± 0.006, COLL − 0.0001 ± 0.006, WHEY − 0.0049 ± 0.01) (*PP, mean[%∙h*^*−1*^*]* ± *SEM:* CARB 0.0094 ± 0.008, COLL 0.0013 ± 0.006, WHEY − 0.0032 ± 0.011) (Fig. 2a, b). Comparing the ΔΔ FSR between groups in the training arm, no difference was observed for the mITT analysis (*p* = 0.98) (m*ITT, mean[%* ± *SEM]:* WHEY − 0.0049 ± 0.01, LITW − 0.0022 ± 0.009, HRTW − 0.0039 ± 0.009 (Fig. 2c). The PP analysis was not possible to perform in the training arm due to the low number of participants fulfilling the PP criteria (LITW: *n* = 4; HRTW: *n* = 3). To take advantage of the unique power of the data set, we explored the 0-month time point by performing a two-way ANOVA test. The effect of feeding (comparing basal with response period) appeared significant (*p* = 0.030), whereas the factor sex did not appear significant (*p* = 0.23) nor did the interaction (*p* = 0.47). However, a closer look at the sex-dependent responses revealed that the overall feeding effect was carried the by the responsiveness in females (FSR_basal_ 0.035 ± 0.002; FSR_response_ 0.041 ± 0.002; paired *t* test *p* = 0.0002) and not males (FSR_basal_ 0.034 ± 0.002; FSR_response_ 0.037 ± 0.002; paired *t* test *p* = 0.16), Fig. 2d, e. Further, we did not find any difference in basal FSR between males (*n* = 37) and females (*n* = 29) (unpaired *t* test, *p* = 0.75; males: 0.034 ± 0.002; females: 0.035 ± 0.002, mean ± SEM). The plasma tracer steady-state enrichment and plasma amino acid concentration during the acute trial can be found in supplemental 2.

### Muscle metabolome

**Fig. 3:**
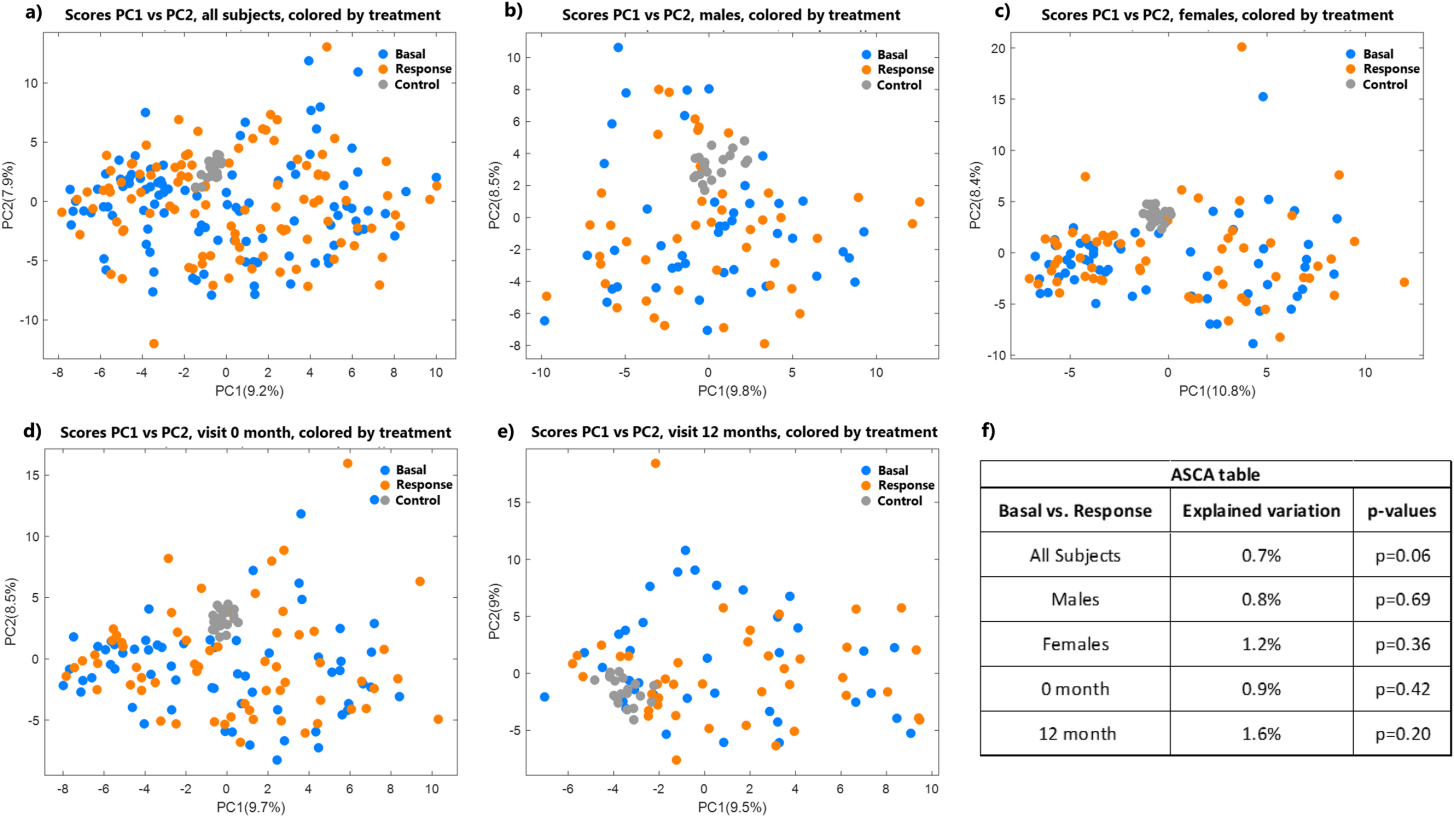
PCA model of the muscle metabolome. Scores for PC1 vs PC2 colored according to basal vs response, for all subjects (**a**), males (**b**) and females (**c**) at both visits, and all subjects at visit 0 month (**e**) and 12 months (**d**). 25% of variation is captured by the first three principal components of the PCA model, although no trend of separation of samples was observed according to treatment irrespective of sex or visit. Control samples are grey and clustered well in all plots. ASCA results according to basal vs response are presented in the table **(f)**

The metabolite data of the skeletal muscle metabolome contained 191 peaks resolved from the GC–MS data. From these, 74 metabolites were identified at level 2 based on Metabolomics Standards Initiative [29], and identification criteria were set to EI-MS match of ≥ 750, RI match of ± 50 and metabolites with labile protons being trimethylsilylated (TMS). These metabolites included 17 amino acids, 12 fatty acids, 11 carbohydrates, 9 organic acids, 7 sugar alcohols, 3 phenolics, and 10 other metabolites including 2 indole derivatives, ibuprofen, uric acid, and cholesterol (supplemental 1). A PCA of the metabolite table shows that up to 25% of the variation is captured by the first three principal components. However, no trend of separation of samples was observed according to visit, treatment, sex, or the CALM intervention design (supplemental 3). In support of this, the ASCA analysis also revealed no effect of the treatment, i.e., metabolome at 0 min did not differ from the metabolome at 240 min after the ingestion of 20 g of whey hydrolysate and 10 g of sucrose (*p* = 0.06) (Fig. 3f), nor was there any separation or significant difference in the overall muscle metabolome due to the treatment even when evaluated separately for the two different visits (0 month and 12 months) and sex separately (males and females) (Fig. 3). Univariate analysis using one-way ANOVA revealed two metabolites, 3-hydroxybutyric acid and 2-butenedioic acid, which were found to be significantly lower at 240 min at both visits (3-hydroxybutyric acid: 0 m: *p* = 0.0025, effect size = 13.8%, *n* = 61; 12 m: *p* = 0.019, effect size = 18.1%, *n* = 39; 2-butenedioic acid: 0 m: *p* = 0.0025, effect size = 14.7, *n* = 61; 12 m: *p* = 0.049, effect size = 14.8%, *n* = 39). Similarly, ASCA analysis revealed no significant effect in relation to the visit (basal 0 month versus basal 12 months) (*p* = 0.62, *n* = 61) (supplemental 4a). Furthermore, ASCA analysis showed no difference between the intervention groups at 12 months (*p* = 0.68, CARB *n* = 6, COLL *n* = 6, WHEY *n* = 6) (supplemental 4b).

## Discussion

This study investigated the 1-year impact of supplementation with proteins alone or in combination with resistance exercise on basal overnight fasting and 4-h postprandial skeletal muscle protein synthesis rate and muscle metabolome profile.

No impact of the protein supplementation arms was found on the muscle protein synthesis rate. Similarly, 1-year exercise with protein supplementation did not seem to affect the muscle protein synthesis rate in comparison to protein supplementation alone. Further, this study confirms the possibility of measuring the metabolome using a low amount of muscle tissue sample (~ 25 mg) using an untargeted GC–MS based metabolomics approach.

With respect to the 1-year intervention arms, we did not find any effect on the FSR responses, which was in contrary to our hypothesis and despite that participants in both COLL and WHEY significantly increased their protein intake [20]. The present data reveal that after a long time allowing a new metabolic steady state to occur, enhanced protein intake seems to have no impact on basal and postprandial muscle FSR. To our knowledge, no studies have previously investigated such effect in a long-term setting. One should, however, due to the relatively high dropout rate, be cautious making firm conclusions. Nonetheless, the results in this study support the results from Højfeldt et al. [10] as well as Gorissen et al. [30], where the acute response in elderly men to a standardized meal and 25 g whey protein after being habituated to a high protein diet for 3 weeks and 2 weeks did not alter the FSR in the acute response period. Further, the study by Højfeldt et al. [9] actually showed an impaired whole-body response indicating an adaptation to the high protein, making the body less sensitive to stimulation of protein synthesis by oral protein intake. Gorissen et al. [30] also showed that habituation to a low compared to a high protein diet actually increased the postprandial release of dietary protein-derived amino acids in the circulation in the low protein group by reducing the splanchnic extraction, thereby allowing more of the dietary protein-derived amino acids to be available to the periphery. Overall, one should be very cautious extrapolating the results from these types of studies into any kind of recommendation, since even very small changes in MPS and imbalances in net balance could have a high impact on muscle mass in the long term.

As for the basal muscle FSR, we did not find any sex differences: unpaired *t* test, *p* = 0.75, males (*n* = 37) and females (*n* = 29). Only few studies have investigated differences between sexes in elderly adults. Henderson et al. [31] found a difference in the basal FSR between males and females in old adults (basal FSR:females > males, Age: ≥ 60 years, *n* = 87), Hirsch et al. [32] found a difference in the basal FSR between males and females (basal FSR: females > males, age: 18–35 years and 51–81 years, *n* = 146) and Smith et al. [33] found a difference in basal FSR between elderly (basal FSR: females > males, age: 65–80 years) males (*n* = 13) and females (*n* = 16). This discrepancy between findings could be explained by different study populations, since it has been shown that adiposity is associated with increased whole-body protein metabolism [34] and that the BMI of the participants in the referenced studies was somewhat higher (BMI: ~ 38 kg m^−2^ [33], ~ 26 kg m^−2^ [31] ~ 28 kg m^−2^[32]) than that of the participants in this study (~ 25 kg m^−2^). Further, the inclusion criteria in the present study were quite specific in terms of habitual physical activity and exercise training, and maybe the similarity of activity levels in our population explains the evenness compared to previous studies. Otherwise, we have no other suggestions for the diversity in findings, but would refer to a high statistical power in the present comparison, which emphasizes the validity of the present results and that it is very unlikely that there is a sex difference on the basal muscle FSR in our population of healthy older adults.

Acknowledging the sparse data on sex differences on the postprandial responsiveness to protein intake, we performed a *t* test at baseline FSR despite seeing no interaction in the ANOVA for explorational purposes. This comparison revealed that only females had an increased FSR in response to protein intake, which is in accordance with Horstman et al. [35]. However, females in this study received significantly (unpaired *t* test, *p* < 0.0001) more protein per kg LBM than males (females, *n* = 29: 0.50 ± 0.05 g kg^−1^ LBM; males, *n* = 36: 0.36 ± 0.04 g kg^−1^ LBM; [mean ± SEM]). However, when performing correlations between protein per kg LBM and FSR response for both sexes separately, we found no link (males *p* = 0.48 and females *p* = 0.50). Thus, our data indicate an actual sex difference between the muscle FSR responses to protein intake in the acute trials.

Human muscle metabolome data have to our knowledge only been presented in three previous studies [14, 15, 36]. Fazelzadeh et al. [14] found 96 separate metabolites using targeted analytical platforms (UPLC–MS/MS, GC–MS) in young (*n* = 30 males) and healthy (*n* = 47 m/19f) and frail (*n* = 25 m/18f) older adults. Sato et al. [15] found 625 different metabolites by combining GC–MS and UPLC–MS/MS in seven overweight (BMI > 27) middle-aged males, and Saoiet al. [36] found 84 metabolites using untargeted MSI-CE-MS. Differently from previous reports, our baseline sample included a larger number of participants (*n* = 65 (36 m/29f)) and we detected 191 peaks from the untargeted GC–MS approach. Of these, 74 muscle metabolites were assigned at level 2 according to the proposed minimum reporting standards for chemical analysis suggested by CAWG and MSI [29] and many more remained unknown due to a limited number of studies performed on this biological sample. Further studies are required for their identification. Interestingly, we did not find any difference in the metabolomics data between the fasting biopsy and the biopsy taken 240 min after protein and glucose intake, with the result being consistent for both sexes at both 0 and 12 months. We observed a significant increase in the plasma AA concentrations after the supplement intake. Plasma AA concentrations peaked after ~ 60 min and had almost declined to fasting levels at 240 min (supplemental 2). We therefore ascribe the absence of metabolome differences between overnight fasting and 240-min postprandial to the fact that any impact of the supplement to the muscle metabolome had faded out and returned to basal level. The only alteration of the muscle metabolome observed was the decrease in concentration of the two metabolites, 3-hydroxybutyric acid and 2-butenedioic acid at the 240-min time point biopsy, which was a consistent finding at both 0 and 12 months. 3-Hydroxybutyric acid is a ketone produced by the liver and used in extrahepatic tissue during fasting or glucose deprivations [37]. Keeping in mind that the subjects had been fasting for ~ 15 h when receiving the protein and glucose drink, which contained only ~ 510 kJ corresponding to ~ 6% of their estimated total energy requirement [38], it is plausible that a substantial part of the nutrients supplied had been metabolized long before the 240-min postprandial time point. Unfortunately, we cannot report on what happened earlier in the postprandial period due to the absence of more frequent biopsies. To investigate the impact of intake of supplements and meals on muscle metabolome, future studies should be designed with more frequent sampling earlier on in the postprandial period. Despite participant attrition and dropout, the important power of the muscle metabolome data is obvious. The available data are at this stage relevant to science in nutrition and physiology as it presents novel reference data as well as methodology within the largely unexplored area of the skeletal muscle metabolome. Further, the data presented are unique with respect to sex comparison, since the current available literature is primarily based on studies including mainly males.

### Limitations

The main limitation in this study is that the number of participants completing the 12-month acute trial with an acceptable adherence to the intervention was lower than expected. We were therefore not able to conduct a PP analysis on the FSR in the training arm as originally planned. Further, it has recently been pointed out that there could be issues regarding recycling of tracer for the FSR calculations, which could impact the results when conducting repeated tracer infusion trials despite them being separated by several months [39]. In this study we observed a larger variation in the basal period at 12 months, which could be caused by the tracer recycling in the structural proteins at 12 months. Shifting to another label on the traced amino acid is recommended in future studies. Lastly, we only evaluated the fasted and 4-h response to feeding in the acute trial at 0 and 12 months. Despite the high sensitivity of the tracer methodology applied, it is still possible that we have missed a cumulative effect of continuedly protein intake that could attenuate the loss of muscle.

## Conclusions

One year of daily protein or carbohydrate supplementation neither seemed to alter the basal nor the protein-stimulated postprandial muscle protein synthesis rate in healthy older adults. In contrast to previous findings, no differences in basal protein synthesis rates between males and females were observed. The applied untargeted GC–MS methodology for measuring the skeletal muscle metabolome was efficient and robust and yielded a relatively high number of metabolites in comparison to the previous studies despite the use of only one analytical platform. The simple sample preparation protocol and the low amount of tissue required to perform the analysis make it a promising method for future investigation of the skeletal muscle metabolome.

## Supplementary Information

Below is the link to the electronic supplementary material.

Supplementary file1 (PDF 634 KB)

## Data Availability

Data described in the manuscript, code book, and analytic code will be made available upon request to the corresponding author.
