# Supplementary material for: Effect of 1-year daily protein supplementation and physical exercise on muscle protein synthesis rate and muscle metabolome in healthy older Danes: a randomized controlled trial
Source: Eur J Nutr. 2023 Jun 2;62(6):2673–85. doi: 10.1007/s00394-023-03182-0 (PMC10421766; doi:10.1007/s00394-023-03182-0)

Supplemental 1. List of metabolites identified from the GC-TOF-MS metabolomics of muscle samples. All metabolites are tentatively identified at level based on the Metabolomics Standards Initiatives[36]. *Occurrence represent the presence of the metabolite peak among the analyzed samples.

**No.**	**Metabolite**	**Retention index**	**Retention time (sec)**	**Occurance (%)**
1	formamide	1000.00	412.41	75
2	acetamide	1000.00	414.82	54
3	un1	1000.00	415.01	72
4	un2	1000.00	415.76	56
5	sulfide	1000.00	418.54	98
6	un3	1000.00	420.05	99
7	un4	1000.00	420.83	54
8	un5	1010.72	430.57	98
9	un6	1012.94	432.28	100
10	mercaptoacetic acid	1015.00	433.87	96
11	un7	1016.59	435.10	92
12	hexanol-1	1019.44	437.29	69
13	un8	1024.17	440.95	81
14	un9	1024.97	441.57	34
15	un10	1028.75	444.48	60
16	1,2-butanediol	1029.83	445.32	71
17	un11	1032.38	447.29	90
18	un12	1033.30	448.00	60
19	un13	1035.97	450.06	56
20	un14	1038.44	451.97	85
21	un15	1043.11	455.57	57
22	un16	1046.45	458.15	93
23	un17	1057.63	466.79	56
24	limonene	1059.86	468.51	98
25	un18	1065.08	472.54	59
26	un19	1066.77	473.84	100
27	lactic acid 1	1067.41	474.34	38

28	un20	1074.11	479.51	44
29	lactic acid 2	1076.05	481.01	99
30	oxalic acid	1079.78	483.89	99
31	un20	1082.77	486.19	69
32	un21	1083.25	486.56	92
33	hexanoic acid	1088.86	490.89	77
34	un22	1093.16	494.22	50
35	un23	1098.48	498.32	39
36	un24	1106.21	504.29	83
37	oxalic acid 2	1114.41	510.62	39
38	alanine	1114.68	510.83	91
39	un25	1118.29	513.62	41
40	glycine 1	1131.47	523.79	76
41	un26	1133.75	525.55	99
42	un27	1141.64	531.65	100
43	un28	1146.77	535.60	34
44	un29	1148.25	536.75	98
45	leucine 1	1164.34	549.17	43
46	3-hydroxybutanoic acid	1166.13	550.55	82
47	n-amylbenzene	1170.95	554.27	60
48	un30	1175.15	557.52	99
49	2-aminobutyric acid	1177.16	559.07	42
50	un31	1180.89	561.95	57
51	Phosphoric acid, monomethyl ester	1182.02	562.82	94
52	un32	1184.32	564.59	58
53	un33	1191.02	569.77	89
54	un34	1194.78	572.67	45
55	un35	1196.72	574.17	52

56	un36	1200.50	577.05	77
57	un37	1204.26	579.64	91
58	un38	1211.29	584.49	100
59	un39	1215.87	587.65	99
60	valine	1218.75	589.64	69
61	un40	1220.09	590.56	100
62	un41	1223.69	593.05	51
63	un42	1229.35	596.95	97
64	un43	1238.91	603.55	67
65	benzothiazole	1241.92	605.62	50
66	diethylene glycol	1246.06	608.48	79
67	benzoic acid	1252.11	612.66	100
68	serine	1260.19	618.23	81
69	octanoic acid	1267.56	623.32	94
70	phosphoric acid	1275.93	629.09	98
71	un44	1279.78	631.75	61
72	threonine	1297.16	643.74	79
73	glycine 2	1309.13	652.00	36
74	un45	1310.10	652.67	54
75	un46	1311.07	653.34	42
76	succinic acid	1315.21	656.19	100
77	un47	1330.13	666.49	91
78	un48	1337.13	671.32	36
79	un49	1341.60	674.40	96
80	un50	1347.04	678.15	60
81	maleic acid	1350.92	680.83	88
82	un51	1353.11	682.35	47
83	un52	1356.81	684.90	99
84	un53	1359.76	686.94	88
85	nonanoic acid	1361.74	688.30	100
86	un54	1370.39	694.27	100
87	un55	1379.93	700.85	48
88	un56	1390.25	707.97	97

89	un57	1391.31	708.71	62
90	un58	1396.64	712.38	98
91	un59	1398.66	713.78	56
92	aspartic acid	1422.79	728.97	39
93	un60	1441.41	740.63	100
94	decanoic acid	1457.97	751.00	90
95	un61	1474.03	761.06	91
96	un62	1487.26	769.34	92
97	un63	1491.85	772.22	65
98	3,4-dimethylbenzoic acid	1493.79	773.43	57
99	4-hydroxybenzoic acid, methyl ester	1496.04	774.84	61
100	un64	1509.19	783.08	52
101	un65	1520.04	789.87	99
102	un66	1530.60	796.48	78
103	un67	1537.73	800.95	37
104	un68	1539.56	802.09	96
105	un69	1549.53	808.34	90
106	un70	1557.55	813.36	39
107	un71	1564.47	817.69	100
108	un72	1589.01	833.06	100
109	un73	1605.03	842.75	94
110	benzothiazole, 2-(methylthio)-	1614.70	848.15	94
111	un74	1632.20	857.92	40
112	un75	1634.39	859.14	57
113	lauric acid	1650.64	868.21	92
114	un76	1662.25	874.70	99
115	un77	1673.41	880.92	97

116	un78	1693.16	891.95	99
117	un79	1703.13	897.52	86
118	un80	1733.92	914.71	58
119	un81	1759.28	928.87	90
120	un82	1826.59	965.08	65
121	tetradecanoic acid	1846.50	975.16	98
122	un83	1849.03	976.45	89
123	un84	1853.41	978.67	73
124	un85	1861.90	982.97	38
125	un86	1878.00	991.12	99
126	glucose 1	1890.42	997.42	40
127	un87	1914.77	1009.76	50
128	ribitol	1926.61	1015.76	91
129	glucose 2	1935.52	1020.27	100
130	un88	1956.71	1031.01	34
131	caproamide	1971.24	1038.37	38
132	glucose 3	1974.72	1040.14	42
133	un89	1999.41	1052.65	74
134	cis-9-hexadecenoic acid	2021.48	1062.90	58
135	inositol	2025.06	1064.56	99
136	hexadecenoic acid	2044.39	1073.50	100
137	1-undecene, 11-nitro	2081.95	1090.90	36
138	myoinositol	2090.52	1094.87	100
139	un90	2125.88	1111.24	95
140	un91	2145.62	1120.38	71
141	un92	2167.10	1130.33	80
142	un93	2172.20	1132.69	75
143	un94	2179.82	1136.22	71
144	linoleic acid	2207.72	1148.84	93
145	oleic acid 1	2214.46	1151.71	100
146	oleic acid 1	2220.75	1154.38	74
147	stearic acid	2241.73	1163.29	100
148	un95	2287.85	1182.90	83

149	un96	2302.60	1189.17	38
150	un97	2315.85	1194.80	35
151	un98	2336.86	1203.73	86
152	oleic acid amide	2364.49	1215.47	80
153	un99	2377.56	1221.02	40
154	hexanedioic acid, bis(2-ethylhexyl) ester	2389.19	1225.97	99
155	un100	2500.68	1239.99	77
156	un101	2596.07	1248.93	85
157	un102	2617.76	1257.39	63
158	un103	2620.58	1258.67	55
159	un104	2622.48	1259.54	53
160	un105	2632.08	1263.91	88
161	un106	2664.96	1278.89	76
162	un107	2674.82	1283.38	85
163	1-monopalmitin	2687.54	1289.17	96
164	un108	2698.85	1294.33	44
165	un109	2706.24	1297.69	71
166	un110	2721.48	1304.63	98
167	un111	2748.61	1316.99	90
168	un112	2753.01	1319.00	37
169	un113	2764.69	1324.32	86
170	un114	2769.60	1326.55	52
171	un115	2773.24	1328.21	67
172	un116	2776.90	1329.88	42
173	un117	2779.78	1331.19	77
174	2,3-dihydroxy-stearic acid, propyl ester	2788.08	1334.97	84
175	dodecanamide	2793.22	1337.31	97
176	un118	2800.94	1340.60	35
177	squalene	2818.10	1344.16	100
178	un119	2820.53	1344.66	78
179	un120	2865.84	1354.07	75

180	un121	2899.34	1361.03	41
181	un122	2915.52	1364.39	47
182	un123	2945.01	1370.51	85
183	un124	2964.86	1374.63	64
184	un125	2988.13	1379.46	67
185	un126	3066.61	1397.64	72
186	un127	3096.48	1404.68	34
187	un128	3103.13	1406.25	52
188	un129	3143.68	1415.82	51
189	un130	3153.74	1418.19	94
190	cholesterol	3174.90	1423.18	100
191	un131	3582.30	1561.54	96

Supplemental 2. Total plasma amino acid concentrations at timepoint 0, 20, 40, 60, 90 and 240 min at 0 and 12 months. No effect of groups was observed at 0 month (p=0.58) or 12 months (p=0.44). * denotes significantly different from 0 min, p<0.0001. Analyzed using mixed effects analysis with Dunnets multiple comparison test in GraphPad Prism v. 8.0.0. TTR at timepoint -180, 0, 20, 40, 60, 90 and 240 min at 0 and 12 months. No effect of groups was observed at 0 month (p=0.28) or 12 months (p=0.54). * denotes significantly different from -180 min, p<0.0001. Analyzed using mixed effects analysis with Dunnets multiple comparison test in GraphPad Prism v. 8.0.0.

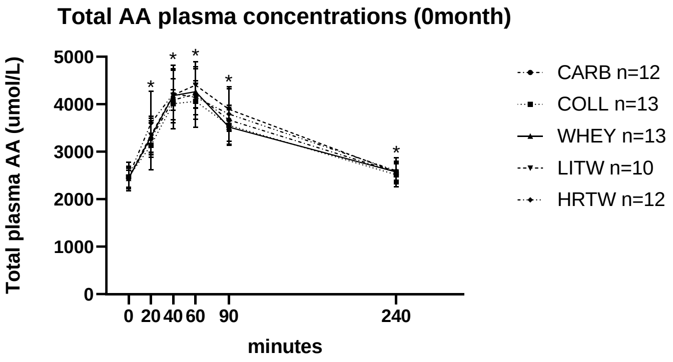

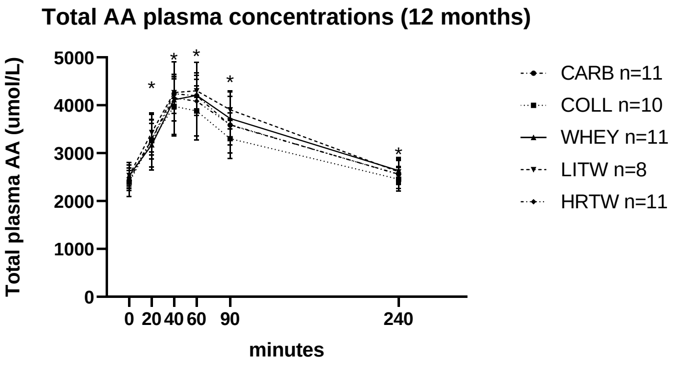

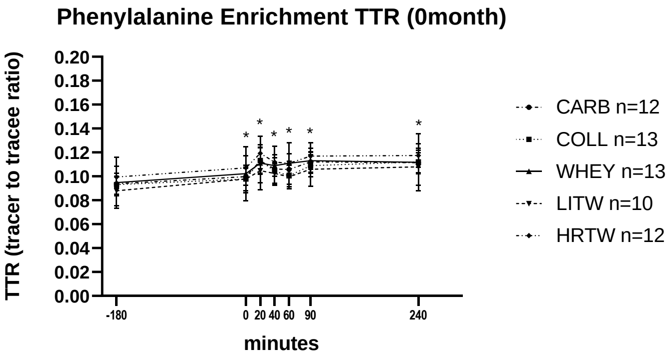

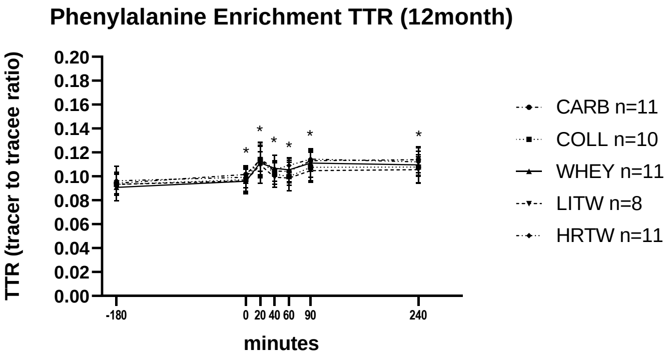

Supplemental 3. PCA model of the muscle metabolome. Scores and loadings for PC1 vs PC2 colored according to baseline vs 12months, basal vs response, sex and CALM design (4th column). 25% of variation is captured by the first three principal components of the PCA model, although no trend of separation of samples was observed according to visit, treatment, sex or the CALM intervention design. Control samples are grey and clustered well in all plots.

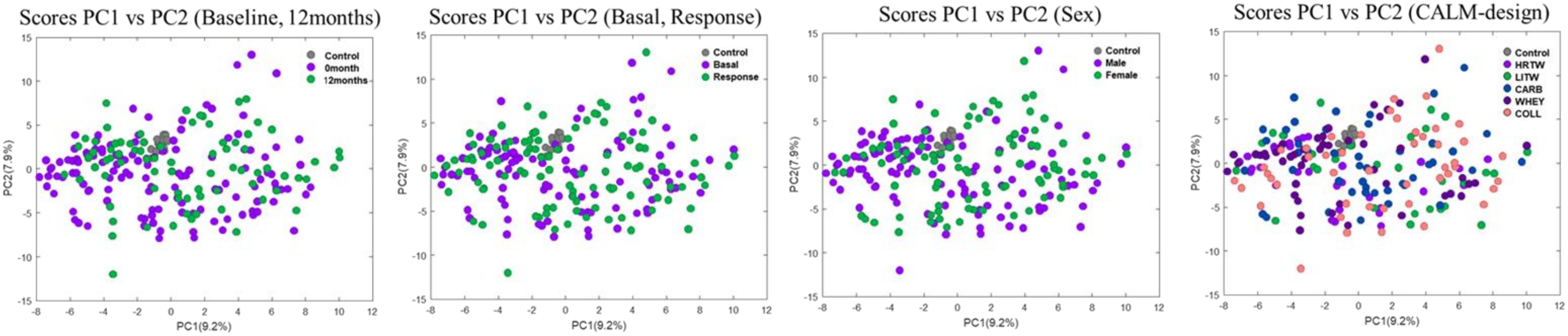

Supplemental 4. ASCA plots**. A)** Scores SC1 colored according to basal at 0 and 12 months (p=0.62). **B)** Scores SC1 vs SC2 of basal at 12 months nutritional arm only colored according to CALM study design (ASCA: p=0.68)

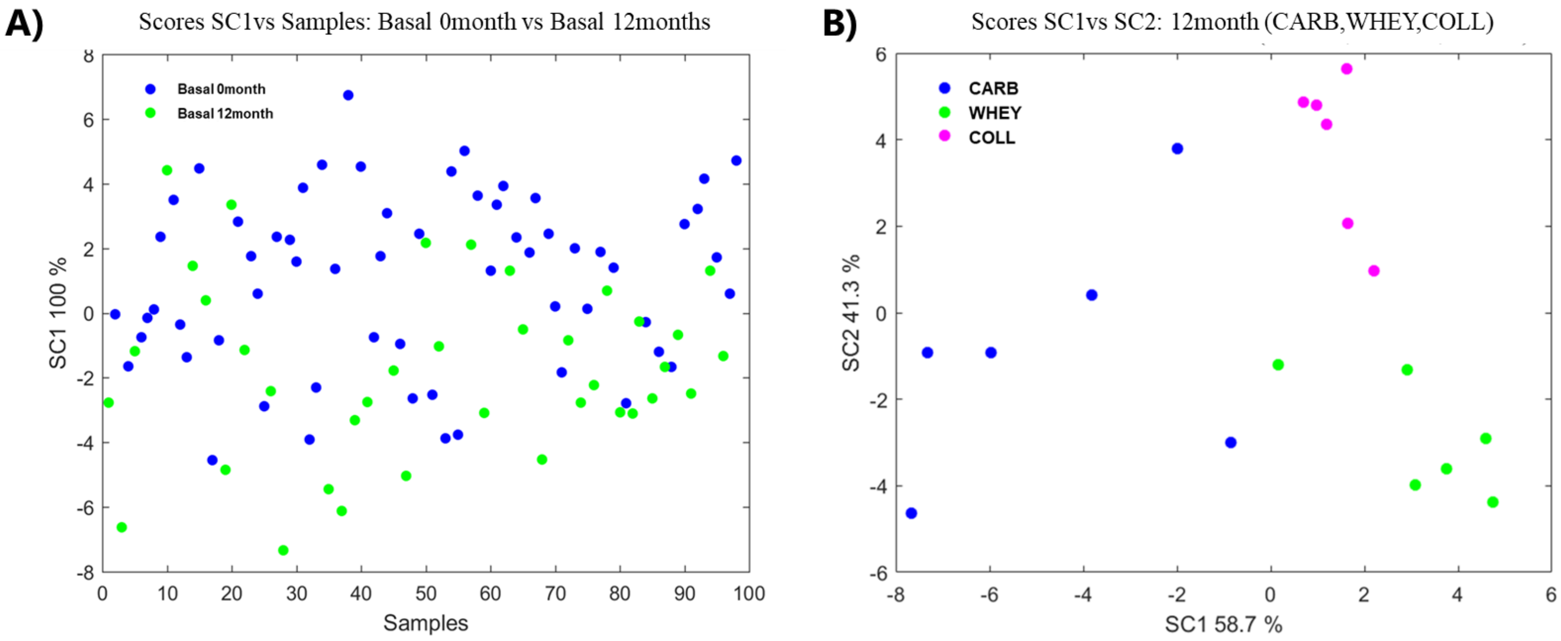

Supplemental 5. CONSORT diagram

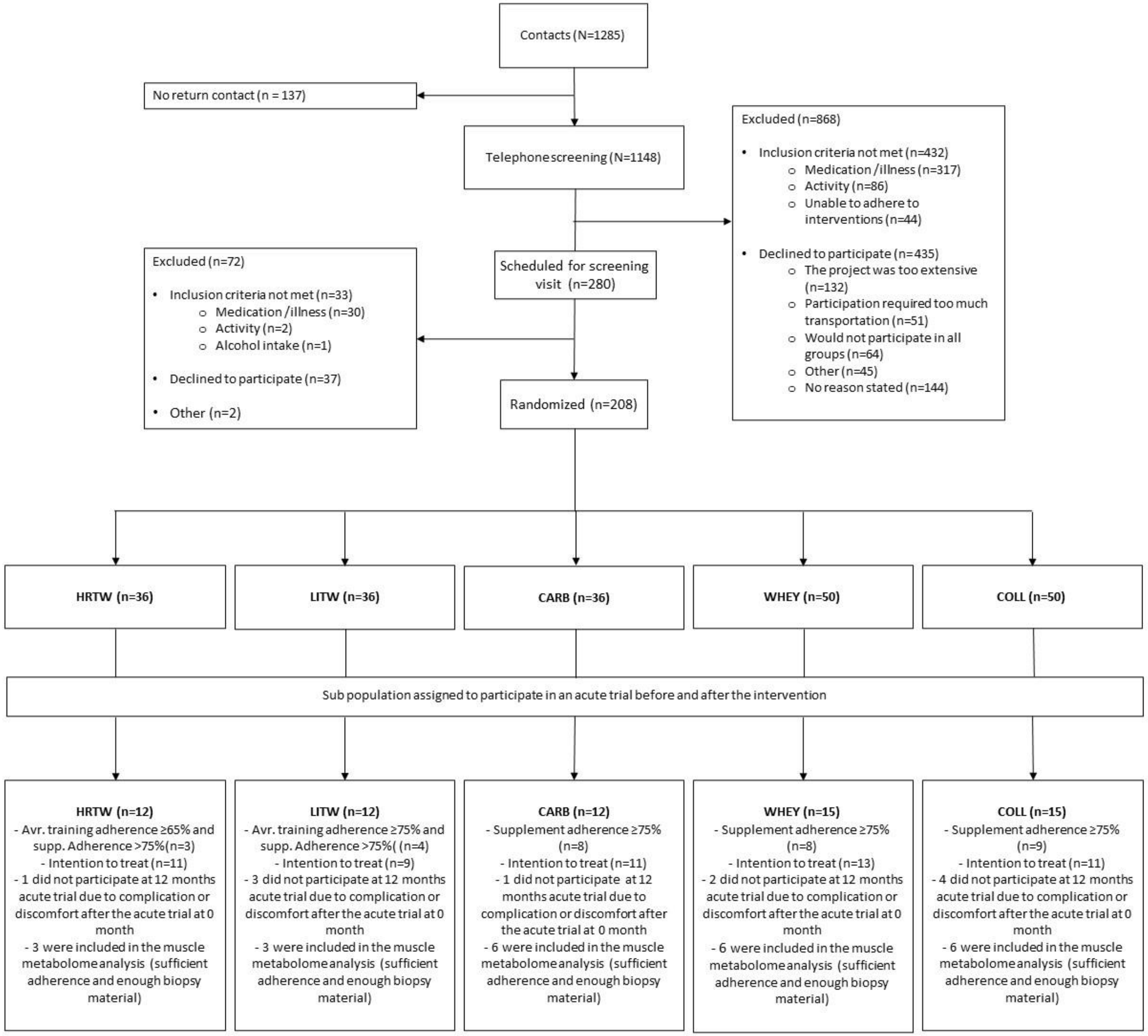

Supplement: Supplementary file 1 — Supplementary file1 (PDF 634 KB) [file 394_2023_3182_MOESM1_ESM.pdf]
